# Inappropriate Antibiotic Use in Zimbabwe in the COVID-19 Era: A Perfect Recipe for Antimicrobial Resistance

**DOI:** 10.3390/antibiotics11020244

**Published:** 2022-02-13

**Authors:** Itai Chitungo, Tafadzwa Dzinamarira, Tinashe K. Nyazika, Helena Herrera, Godfrey Musuka, Grant Murewanhema

**Affiliations:** 1 Chemical Pathology Unit, Department of Medical Laboratory Sciences, Faculty of Medicine and Health Sciences, University of Zimbabwe, Harare, Zimbabwe; ichitungo@medsch.uz.ac.zw; 2 School of Health Systems & Public Health, University of Pretoria, Pretoria 0002, South Africa; gm2660@cumc.columbia.edu; 3 ICAP at Columbia University, Harare, Zimbabwe; 4 Department of Clinical Sciences, Liverpool School of Tropical Medicine, Liverpool L3 5QA, UK; tknyazika@gmail.com; 5 School of Pharmacy and Biomedical Sciences, University of Portsmouth, Portsmouth PO1 2UP, UK; helena.herrera@port.ac.uk; 6 Unit of Obstetrics and Gynaecology, Department of Primary Health Care Sciences, Faculty of Medicine and Health Sciences, University of Zimbabwe, Harare, Zimbabwe; gmurewanhema@yahoo.com

**Keywords:** COVID-19, antimicrobial resistance, antimicrobial stewardship

## Abstract

The global COVID-19 pandemic has resulted in an upsurge in antimicrobial use. The increase in use is multifactorial, and is particularly related to the empirical treatment of SARS-CoV-2 and suspected coinfections with antimicrobials and the limited quality of diagnostics to differentiate viral and bacterial pneumonia. The lack of clear clinical guidelines across a wide range of settings, and the inadequacy of public health sectors in many countries, have contributed to this pattern. The increased use of antimicrobials has the potential to increase incidences of antimicrobial resistance, especially in low-resource countries such as Zimbabwe already grappling with multidrug-resistant micro-organism strains. By adopting the antimicrobial stewardship principles of the correct prescription and optimised use of antimicrobials, as well as diagnostic stewardship, revamping regulatory oversight of antimicrobial surveillance may help limit the occurrence of antimicrobial resistance during this pandemic.

## 1. Introduction

The ongoing battle with coronavirus disease 2019 (COVID-19) started when it was first designated a pandemic in March 2020. The pandemic has presented unprecedented challenges for the population, with over five million confirmed fatalities from the disease globally over two years [1]. As COVID-19 is a novel virus whose treatment strategies were unknown at the onset, one of the most significant and ongoing challenges that initially emerged was the use of unauthorised, substandard, and falsified prevention and treatment pharmacological packages supplied via formal and informal channels. The lack of clear clinical guidelines across a wide range of settings, and the inadequacy of public health sectors in many countries, have contributed to this pattern. The pandemic has, as a result, witnessed a surge in antimicrobial prescription and use, which has resulted in a growing concern for a potential rise in antimicrobial resistance (AMR) [2]. This comes at a time when the World Health Organisation (WHO) has brought to the fore the threat of AMR and has renewed its calls for antimicrobial stewardship (AMS) to preserve the antimicrobials, which are currently still effective for future generations [3].

The discovery of antibiotics in the early 20th century significantly improved the treatment and management of bacterial infections. Over the subsequent years, there was a rapid expansion in the range of not only the antibacterial agents, but also the other antimicrobial agents available to treat different kinds of infection, with access to them increasing for the general population. The availability of effective antimicrobial agents, especially in sub-Saharan Africa, where the burden of infectious diseases is high, remains critical for adequate patient outcomes. However, over the years, antimicrobials, and in particular antibiotics, have prevailed as the most prescribed medications for patients, when their use has not been appropriate [4]. Both the overuse and improper use of antibiotics (including in humans, livestock, and food production), poor infection prevention and control practices, and international travel are considered some of the leading drivers for the emergence and spread of AMR [5].

The negative consequences of increasing AMR to global health are well documented and multiple, and include limiting the treatment options for resistant pathogens responsible for significant burdens of disease. For example, multidrug-resistant tuberculosis (MDR-TB) has emerged, requiring more antibiotics, and with increased toxicity to patients and high costs. In the case of tuberculosis, even extreme drug-resistant TB (XDR-TB) has been documented, which has serious global health implications [6]. A recently published systematic analysis of the global burden of bacterial AMR in 2019 highlights the magnitude of the problem. Based on predictive mathematical models, an estimated 4.95 million (95% CI 3.62–6.57 million) deaths were associated with bacterial AMR in that year, with 1.27 million (0.911–1.71) attributable to AMR. The highest burden of all-age death rates attributable to AMR is estimated to occur in the western sub-Saharan Africa region, at 27.3 deaths per 100,000 (20.9–35.3). In this way, AMR is killing more people than HIV or malaria, [7] compromising clinical outcomes and leading to an economic impact that cannot be ignored.

AMR results from the natural evolutionary response to antimicrobial exposure and pressure by micro-organisms to acquire the ability to withstand antimicrobial drugs. A key recommendation to fighting AMR is optimising antibiotic use through the correct dosage and administration, ensuring antimicrobial treatment regimens are completed to prevent the survival of the more resistant organisms. Examples of organisms where resistance is widespread have been identified, leading to limited treatment options and serious threats to public health. In the past, significant examples in Zimbabwe have included the reduced effectiveness of antimalarial drugs, which have shifted from chloroquine to sulphadoxine-pyrimethamine to artemisinin-based combination treatments over two decades [8]. Similarly, reports of the resistance of *Neisseria gonorrhoeae* to fluoroquinolones have been noted [9]. The current pattern of antimicrobial prescription and use in Zimbabwe during the COVID-19 era provides a conducive cultural environment to precipitate and propagate AMR, posing a severe threat to the treatment of infectious diseases in the future in a country that already has limited treatment options. Hence, from this viewpoint, we discuss how the ongoing COVID-19 pandemic is contributing to AMR in Zimbabwe, and suggest possible solutions to this global health threat.

## 2. Drivers of Antimicrobial Resistance in the COVID-19 Era

### 2.1. Empirical Treatment for Suspected Bacterial Infections

A previous literature review demonstrated the growing problem of AMR during the COVID-19 pandemic. Potential drivers of AMR in this review were noted as inappropriate antimicrobial exposure, environmental contamination, and the discontinuation of some infection-prevention and control measures. We discuss here some drivers of AMR applicable to Zimbabwe.

COVID-19 patients may receive antimicrobial treatment for several reasons, some of which we highlight here. First, COVID-19 infection has some similarities with bacterial pneumonia. Secondly, the lack of effective SARS-CoV-2 treatment and management guidelines leads to uncertainty, and potentially to the overuse of antibiotics [10]. Lastly, COVID-19 patients may acquire superimposed bacterial infections that require antimicrobial treatment [11,12]. The WHO has published guidelines for the clinical management of patients with COVID-19. However, countries have developed their treatment guidelines for the disease based on resource availability and local infection patterns, contrary to WHO guidelines that advocate that COVID-19 patients are to be administered appropriate antimicrobials after the identification of sepsis [13].

The initial management of COVID-19 was based on early reports from China and modelled using the historical influenza pandemic, which highlighted the potential for a high number of bacterial coinfections [14]. Some studies also indicated that azithromycin had the potential to disrupt viral replication. Zimbabwe, like other nations, drafted COVID-19 treatment and management protocols based on these reports. The absence of a standard treatment regimen for COVID-19, combined with the lack of laboratory facilities for testing in resource-limited countries, caused some nations to adopt empirical approaches to antimicrobial prescription and use, leading to the rampant use of antimicrobials such as azithromycin, doxycycline, and ceftriaxone to treat and manage COVID-19 patients [6,13,15]. At the same time, the use of antibiotics such as ceftriaxone for patients with severe COVID-19 who have presumptive superimposed bacterial infections found its way into national treatment guidelines [15,16]. A rapid review of COVID-19 treatment guidelines across 10 African countries, including Zimbabwe, noted that a wide range of antimicrobials was included for use without a solid scientific basis [15]. Similarly, antiparasitic and disease-modifying agents, such as hydroxychloroquine and ivermectin, have been widely used, whilst antivirals have also been prescribed indiscriminately in some settings [16,17]. This irrational pattern of use has led to fears of the emergence and propagation of widespread AMR globally. AMR is one of the emerging global health threats of the 21st century; therefore, effective ways of preventing its emergence are urgently called for. This is more so in sub-Saharan Africa, where many diseases of public health significance are infectious in their aetiology [18].

Despite the widespread prescription of antibacterial antimicrobials from the onset of the COVID-19 pandemic, subsequent studies have indicated that, in most cases, less than 5% of the patients had bacterial coinfections. The International Severe Acute Respiratory and Emerging Infections Consortium (ISARIC) reports that about a third of COVID-19 treatments include antimicrobial drugs, set against a 7 to 8% bacterial or fungal coinfection rate based on results from meta-analyses [6,19].

### 2.2. Limited Diagnostic Capacity

Laboratory diagnosis is essential to distinguish between viral and bacterial pneumonia. Challenges in accessing COVID-19 testing, or prolonged turnaround times to differentiate between COVID-19 and bacteria pneumonia, lead to empirical treatment without the determination of the aetiology of the disease. Initially, COVID-19 real-time polymerase chain reaction (RT-PCR) testing was not easily accessible in resource-limited settings. Despite Zimbabwe’s move to cheaper rapid antigen detection test kits, these are not always readily available, accessible, or affordable. Additionally, COVID-19 patients may acquire secondary bacterial coinfections which require antimicrobial treatment [11]. This is more so in severely ill, hospitalised, or bed-ridden patients who may easily acquire nosocomial infections. Correct diagnosis of the infecting micro-organisms is critical for the administration of the appropriate antimicrobial treatment, mainly as some nosocomially acquired microbes might already have developed resistance to a wide range of antibiotics, an example being methicillin-resistant *Staphylococcus aureus* (MRSA). Microscopy, culture, and sensitivity (MCS) is the generally accepted effective and accurate method of identifying microbial infections in a biomedical laboratory. However, most clinicians adopt empiric treatment for presumed pneumonia [20]. MCS assays for diagnosing bacterial infections and determining the appropriate antibiotics to prescribe are also not readily available in most resource-limited settings, especially at lower levels of healthcare such as clinics, rural hospitals, and district-level hospitals. In Zimbabwe, these assays are generally only available at provincial- and tertiary-level hospitals; however, the current harsh economic situation in the country has led to a shortage of equipment, reagents [21], and appropriately qualified laboratory scientists [22]. In the absence of MCS results, clinicians offer treatment based on their epidemiological knowledge of local infection patterns. Similarly, for patients with severe COVID-19 at risk of prolonged recumbence and superimposed hypostatic bacterial pneumonias, clinicians in some settings empirically prescribe broad-spectrum antimicrobials in the absence of MCS results. Due to fears of AMR by clinicians, some antibiotics such as meropenem, which should only be prescribed based on a supporting culture, are given in the absence of MCS results, leading to reduced treatment options for patients, contrary to AMS principles [6].

### 2.3. Increased Use of Falsified and Substandard Antimicrobials

The pandemic is exacerbating the already proliferating market of falsified or substandard antimicrobials. According to the WHO, substandard products are “authorized medical products that fail to meet quality standards or specifications, or both,” and falsified products are “products that deliberately/fraudulently misrepresent identity, composition, or source.” [23]. Falsified and substandard antimicrobial drugs lead to treatment failure, adverse clinical outcomes, and AMR development due to sub-therapeutic drug dosage [24,25]. Strategies to curb the spread of SARS-CoV-2, such as international travel and trade restrictions at the onset of the COVID-19 pandemic, led to supply chain disruptions and pharmaceutical regulatory governance challenges that created opportunities for the increased influx of falsified, substandard drugs [23,26,27,28]. While the precise market share of informal street markets in low- and middle-income countries is unknown, a study in Nigeria suggested that more than 40% of rural communities consult and purchase drugs from street vendors for illnesses such as childhood fevers [25,29,30]. The use of counterfeit vaccines and antimicrobials leads to the development of new SARS-CoV-2 variants and the emergence of AMR. This is a serious global health threat that needs an urgent discourse at the international level. Thus, strong recommendations and regulatory frameworks must be put in place by individual countries and the WHO to curb the growing markets for falsified or substandard medicines.

### 2.4. Poor Adherence and Compliance to Treatments

The ongoing COVID-19 pandemic has led to disruptions or delays in diagnosis, treatment, or management of chronic infections such as tuberculosis (TB) and the human immunodeficiency virus (HIV) in Zimbabwe [31]. This can potentially lead to selection for drug resistance. Similarly, the disruption of vaccination services may contribute to an increased risk of infections and overuse of antimicrobials [32,33]. Access to testing and care for people living with HIV (PLWH) and TB patients was disrupted due to the restrictive measures imposed to curb the spread of the SARS-CoV-2 virus [34,35]. The disruption to care access and medication for these vulnerable groups can lead to treatment defaults, and potentially the development of resistance. Inclusion of training on AMR for all healthcare workers and subsequent dissemination of such information to communities through various communication channels is an important way of safeguarding against AMR.

### 2.5. Misinformation

A collision of factors during the COVID-19 pandemic has contributed to the spread of misinformation about the disease and the use of antimicrobials. The initial politicisation and underestimation of the pandemic led to inconsistent messaging and confusion among the public. The initial lack of recognition of the existence and characteristics of COVID-19 [36], and the emphasis that the pneumonia-like disease is curable with antibiotics, resulted in an increase in their use, especially azithromycin. This position is more challenging for antimicrobial stewardship (AMS) in low- and middle-income countries (LMICs), which already have a high burden of multidrug-resistant organisms. This situation has not been helped by the rapid dissemination of preprints of poorly designed research studies and the premature or inaccurate media reporting of unsubstantiated findings that often followed. The deluge of misinformation, and limited access to timely, verified, and quality evidence for healthcare professionals primarily in LMICs, exacerbated COVID-19 management controversies. For example, in Zimbabwe, the rampant use of ivermectin has persisted as a contentious issue in the medical community [37].

### 2.6. Antimicrobial Stewardship Deprioritisation

The ongoing pandemic has caused disruption of routine health services, leading to staff and resource redeployments to fight COVID-19 [38]. This has had the effect of overlooking and deprioritising AMS programmes that rely on multidisciplinary collaborations and the regular review of practices [39]. Reduced opportunities to optimse antimicrobial therapy in patients have followed, paired with a lack of expertise in generating evidence-based guidelines for antimicrobial prescribing for COVID-19. Misinformation, alongside anxiety within a medical community facing deteriorating patients [40], has led to increased instances of antimicrobial use and prescriptions, against AMS principles. In Zimbabwe, COVID-19 management was prioritised at the expense of AMS. An example of this is how human resource and testing platforms, such as gene experts for TB, were repurposed to fight the pandemic.

## 3. Strategies to Address Antimicrobial Resistance

### 3.1. Antimicrobial Stewardship

Antimicrobial stewardship (AMS) refers to interventions aimed at improving the rational use of antimicrobials by optimising drug selection, dose, duration, and routes of administration, while minimising toxicity and the conditions for selecting resistant strains [41]. Microbiology laboratories aid AMS by ensuring a quick turnaround for the accurate identification of micro-organisms, and by issuing curated antimicrobial susceptibility reports. Methods for arriving at a correct diagnosis depend on diagnostic stewardship, which optimises sample collection and management by implementing robust quality-assurance processes [42]. The combined strategies of AMS and diagnostic stewardship ensure better patient outcomes (reducing morbidity, mortality, and adverse events), the optimum use of available resources, and the preservation of the clinical effectiveness of antimicrobials by slowing down AMR [43,44]. The ongoing COVID-19 pandemic poses significant threats to AMS activities and is perceived to be a potent driver of AMR.

A combination of proper guideline development and education on the appropriate use of antimicrobials will empower prescribing clinicians and lead to a sustained cultural change in antimicrobial utilisation without compromising patient outcomes and improvements in resistance patterns and costs [4]. Even though the ongoing pandemic is stretching the limits of optimal antibiotic stewardship, a coordinated approach to developing a consensus on the evidence-based guidelines for empirical use of an antimicrobial, and the continued survey of their use by local stewardship teams, are essential to arrest the continued AMR risk [12]. Once national guidelines are promulgated, prescribers must adhere to them during the ongoing COVID-19 pandemic and beyond. For example, Zimbabwe has the Essential Medicines List in Zimbabwe (formerly Essential Drug List in Zimbabwe, EDLIZ), which guides how antibiotics should be prescribed and states the level of care at which they can be used. Patients should also be educated on the appropriate use of antimicrobials, emphasising that these do not treat or cure viral infections such as COVID-19 and influenza [33].

### 3.2. Strengthen Diagnostics and Surveillance

The early diagnosis of SARS-COV-2 infection is crucial to curbing excessive antimicrobial use [12]. Strengthening laboratory diagnostics through capacity building (both laboratory and personnel) is needed to tackle COVID-19 surveillance and guide AMS and diagnostic stewardship, especially at the primary healthcare level in Zimbabwe. With robust laboratory systems in place, the evidence-based prescription and use of antimicrobials will increase, and AMR monitoring and surveillance will become less challenging, informing policy and practice. Countries must take advantage of some of the emerging or improved diagnostics to build back better from the COVID-19 pandemic and help in the fight against AMR.

The surveillance of antibiotic use in the community is essential to inform and evaluate strategies for the prevention and control of AMR [5]. Countries should regularly analyse these data to identify trends, develop and implement standard operating procedures, and design awareness campaigns to improve and optimise antimicrobial use in communities. This should then inform national action plans for the appropriate use of antimicrobial agents, whose effectiveness should be monitored through prospective and continuous surveillance of their resistance. Surveillance data allow for trends and resistance hotspots to be identified, informing strategies to address the arising challenges [19]. Surveillance of AMR in Zimbabwe is currently not as visible; therefore, moving forth, there is a need to strengthen this important aspect, including through the National Microbiology Reference Laboratory and frequent discourses between scientists, clinicians, and public health stakeholders. Resistance testing, where available, should routinely be embedded in surveillance systems for AMR to appropriately inform clinicians on what to prescribe.

### 3.3. Antimicrobial Legislation

AMR is being fought through the development and implementation of regulations and policies. Various countries have enacted legislation to combat antimicrobial prescription, use, and particularly their routine release into the environment at pharmaceutical manufacturing sites as part of the production process. [45]. Robust regulatory frameworks and other measures are critical to curbing the proliferation of falsified and substandard medicines through building national and international regulatory capacity to strengthen the overall regulatory footprint. The African health information technology start-up company RxAll Inc. is testing an innovative approach using a handheld scanner to compare drug spectral scans with data in a cloud database for drug authenticity [23]. The technology, once validated and operational, has the potential to monitor drug supply chains from manufacturing to the point of prescription to the patient, significantly strengthening drug monitoring and regulation

### 3.4. Communication

Most LMICs are overburdened with multidrug-resistant organisms and antimicrobial-use misinformation, combined with a lack of basic knowledge or limited understanding of SARS-CoV-2 infections. This has caused both the public, and in some instances, medical professionals, to have an over-reliance on prescription antimicrobials. A strategy to fight misinformation should be in place and needs to be effective in combating the different routes of misinformation at present, particularly social media. The WHO and the Africa Centre for Disease Control and Prevention already use their digital platforms to optimise antimicrobial use and tackle misinformation by discussing the ineffectiveness of antimicrobials as a primary treatment for COVID-19. Additionally, concerted efforts to rapidly disseminate accurate, timely evidence updates to healthcare workers are essential to the evidence-based management of COVID-19.

## 4. Conclusions

The continued emergence of AMR, worsened by the ongoing COVID-19 pandemic, is an urgent global health priority that needs an urgent discourse at the global level. Concerted, international, multistakeholder approaches to effectively combatting this challenge require commitment and collaboration, as fixing the problem at the individual-country level could be a challenge. Key global health priorities are strengthening AMS activities within and beyond countries and leveraging pre-existing systems, but also taking advantage of improved and emerging diagnostics during the COVID-19 pandemic and beyond. Zimbabwe, as an LMIC with limited capacity for effective AMR, must build international partnerships, especially with research institutions from developed countries and international development partners, to improve its capacity to effectively deal with the ever-growing threat of AMR.

AMR is globally recognised as a growing political concern with profound repercussions for social, economic, human, and animal health. Rebuilding from the COVID-19 pandemic, some of the strategies to address the AMR challenge include issuing clear messages on the pandemic and treatment guidelines to counter misinformation, training of healthcare workers on AMS principles, strengthening AMS through legislation, and capacitation of laboratories to enhance diagnostic stewardship. The collective responsibility for developing and implementing action plans to address antimicrobial use and abuse to avert a public health crisis resulting from AMR is, therefore, a priority.

## Data Availability

Not applicable.
